# Influence of the atmospheric environment on spatial variation of lung cancer incidence in China

**DOI:** 10.1371/journal.pone.0305345

**Published:** 2024-06-18

**Authors:** Haishi Yu, Yang Wang, Xiaoli Yue, Hong’ou Zhang

**Affiliations:** 1 Yunnan Normal University Hospital, Kunming, Yunnan, China; 2 Faculty of Geography, Yunnan Normal University, Kunming, Yunnan, China; 3 Guangzhou Institute of Geography, Guangdong Academy of Sciences, Guangzhou, Guangdong, China; Chiang Mai University, THAILAND

## Abstract

Conducting this research contributes to a deeper understanding of the correlation between atmospheric environmental quality and lung cancer incidence, and provides the scientific basis for formulating effective environmental protection and lung cancer prevention and control strategies. Lung cancer incidence in China has strong spatial variation. However, few studies have systematically revealed the characteristics of the spatial variation in lung cancer incidence, and have explained the causes of this spatial variation in lung cancer incidence from the perspectives of multiple components of the atmospheric environment to explain this spatial variation in lung cancer incidence. To address research limitations, we first analyze the spatial variation and spatial correlation characteristics of lung cancer incidence in China. Then, we build a spatial regression model using GeoDa software with lung cancer incidence as the dependent variable, five atmospheric environment factors—particulate matter 2.5 (PM2.5) concentration, temperature, atmospheric pressure, and elevation as explanatory variables, and four socio-economic characteristics as control variables to systematically analyze the influence and intensity of these factors on lung cancer incidence. The results show that lung cancer incidence in China has apparent changes in geographical and spatial unevenness, and spatial autocorrelation characteristics. In China, the lung cancer incidence is relatively high in Northeast China, while some areas of high lung cancer incidence still exist in Central China, Southwest China and South China, although the overall lung cancer incidence is relatively low. The atmospheric environment significantly affects lung cancer incidence. Different elements of the atmospheric environment vary in the direction and extent of their influence on the development of lung cancer. A 1% increase in PM2.5 concentration is associated with a level of 0.002975 increase in lung cancer incidence. Atmospheric pressure positively affects lung cancer incidence, and an increase in atmospheric pressure by 1% increases lung cancer incidence by a level of 0.026061. Conversely, a 1% increase in temperature is linked to a level of 0.006443 decreases in lung cancer incidence, and a negative correlation exists between elevation and lung cancer incidence, where an increase in elevation by 1% correlates with a decrease in lung cancer incidence by a level of 0.000934. The core influencing factors of lung cancer incidence in the seven geographical divisions of China exhibit variations. This study facilitates our understanding of the spatial variation characteristics of lung cancer incidence in China on a finer scale, while also offering a more diverse perspective on the impact of the atmospheric environment on lung cancer incidence.

## 1. Introduction

Lung cancer has evolved into a major public health challenge globally, posing an immense threat to human well-being and imposing substantial economic and social burdens [1, 2], hindering global sustainable development. According to the National Cancer Center data, in 2015, there were 787,000 new lung cancer patients, leading to 630,000 deaths in China, with 85% of these patients diagnosed at advanced stages. In 2016, China had 820,000 new lung cancer cases, ranking first worldwide, and lung cancer mortality was even higher, reaching 710,000, compared to the previous year [3]. Lung cancer incidence in China has regional heterogeneity; generally, the incidence in East China, North China, and Northeast coastal provinces and cities is significantly higher than in Northwest, Southwest, and South-Central China [4]. However, there are few studies that analyze the characteristics of spatial variation and correlation in lung cancer incidence in China at the county scale. Therefore, it is necessary to study the spatial variation and explore spatial autocorrelation characteristics of lung cancer incidence in China, which will help the government and relevant authorities identify priority areas for lung cancer prevention and treatment in China.

Current research findings indicate that there is a significant spatial heterogeneity in lung cancer incidence. The study conducted by Wei et al. found that lung cancer incidence in Henan Province exhibits significant spatial variability and clustering characteristics. High-incidence hotspots are mainly distributed in Central Henan, Eastern Henan, and Southern Henan, whereas low-incidence cold spots are concentrated in Western Henan, Northern Henan, and some areas of Southern Henan. The study further revealed that the spatial heterogeneity variation is closely related to environmental factors, noting that meteorological conditions, by affecting the state of air quality and the level of exposure to air pollutants, can influence the incidence rates of lung cancer [5]. Similarly, research conducted by Lei et al. on lung cancer incidence in Shenzhen, China, also emphasized spatial differences, finding that the lowest incidence is in the Taoyuan Subdistrict in the Futian District and the highest incidence is in the Dapeng Subdistrict in the Dapeng District [1]. A study conducted in Georgia, USA, found that the risk of lung cancer incidence persistently remained high in the northwestern part of the state from 2000 to 2007 [6]. Research by Camia in Pennsylvania, USA, revealed that during different periods, clusters of high lung cancer incidence are distributed in various regions. For example, from 2010 to 2013, high-incidence clusters were located in the southeastern part of the state, whereas the cluster covering the largest area was found in southwestern Pennsylvania during the same period, including Allegheny, Fayette, Greene, Washington, and Westmoreland Counties [7]. Multiple studies have confirmed significant spatial disparities in lung cancer incidence, reflecting not only the geographic patterns of disease occurrence but also indicating that the quality of the atmospheric environment, socioeconomic status, lifestyle choices, and genetic factors may collectively influence the regional patterns of the disease. Therefore, exploring the driving factors behind the spatial heterogeneity of lung cancer incidence has become a key focus of this research.

Multiple causative factors contribute to lung cancer. In addition to genetic defects (family genetic factors or genetic mutations), some external factors, such as smoking [8, 9], unhealthy lifestyles [10], air pollution [11–13], and specific occupational exposures (ionizing radiation, asbestos, dust, and radon), play a vital role in the cause of lung cancer [14–19] as they can lead to lung lesions. Smoking is a major cause of lung cancer [20, 21], with studies revealing that it accounts for approximately 80% of lung cancer deaths in men and 50% in women globally. Unhealthy lifestyles, such as physical inactivity and consumption of high-calorie foods, are linked to increased lung cancer incidence and mortality [22]. Air pollution causes high concentrations of chemical carcinogens in the air, such as sulfur dioxide (SO_2_), nitrogen dioxide (NO_2_), particulate matter 2.5 (PM2.5), and PM10, resulting in lung lesions and increasing the risk of cancer. Besides, specific occupational exposures, such as ionizing radiation, radon, dust, etc., are demonstrated to affect lung cancer incidence.

Among the causative factors of lung cancer, the atmospheric environment, including air quality (PM2.5, PM10, SO_2_, and NO_2_) [23–25], temperature [23, 26, 27], precipitation [28], atmospheric pressure, and elevation, is a non-negligible cause [29, 30]. For example, Guo et al. discovered that PM2.5 concentration is significantly correlated with adverse effects on lung cancer incidence in men and women [23]. Han et al. confirmed that NO_2_ emerges as the primary environmental factor influencing lung cancer incidence [26]. Lin et al. discovered that the air quality index and annual precipitation levels affect lung cancer, with high incidence seemingly associated with poor air quality and low yearly precipitation [28]. Merrill et al. observed a reduced risk of developing lung cancer and improved patient survival at high altitudes [29]. Additionally, Guo et al. found that temperature appears to positively modify the effect of PM2.5 on lung cancer incidence in men [23]. These studies, to a certain extent, validate the influence of atmospheric environmental factors on the occurrence of lung cancer. However, the atmospheric environment is a complex and integrated system comprising multiple elements such as air quality, temperature, precipitation, atmospheric pressure, and elevation. Moreover, few scholars still study the intensity and direction of the influence of the multi-factor system of atmospheric environment on lung cancer incidence. Different components of the atmospheric environment may exhibit diverse intensity and directions in their impact on lung cancer incidence. Unfortunately, limited attention has been given to this variability among scholars. As a result, there is an urgent need for comprehensive investigations into lung cancer incidence, considering the multifaceted composition of the atmospheric environment.

This study aims to explore the influence of atmospheric environment factors on lung cancer incidence in China. Previous research has highlighted that PM air pollution has become an increasingly severe public health problem in China [31], with PM2.5 identified as the primary pollutant responsible for the frequent occurrence of atmospheric haze, causing air pollution and its deleterious effects on human health. Consequently, we select PM2.5 concentration as an indicator of air quality. Furthermore, we determine the influence of other four atmospheric environment factors on lung cancer incidence, namely temperature, precipitation, atmospheric pressure, and elevation. These environmental factors have received limited attention in previous studies but they are the focal point of our investigation.

With a large population and wide area, China’s geography is complex and varied, exhibiting significant differences in the atmospheric environment among its regions. Given these natural conditions, we take China as an example to explore the influence of the atmospheric environment on lung cancer incidence, which is typical and representative.

We utilize China to establish a conceptual framework for atmospheric environmental factors that significantly impact lung cancer. Firstly, we comply with a cross-sectional dataset encompassing five atmospheric environmental factors and four urban economic and social development factors, covering 2869 Chinese county units. Subsequently, we employ a spatial regression model to investigate the intensity and direction of the influence exerted by PM2.5 concentration, temperature, precipitation, atmospheric pressure, and elevation on lung cancer incidence. This study aims to assist healthcare policymakers in devising targeted strategies and policies for lung cancer prevention, taking into account the unique characteristics of diverse regional atmospheric environments, ultimately aiming to reduce the incidence of lung cancer in high-risk geographical areas within China. Furthermore, the insights gained from this study may serve as supplementary case studies to promote interdisciplinary collaborations between atmospheric sciences, geography, and cancer epidemiology, providing a scientific basis and reference for governments to optimize the allocation of medical resources.

## 2. Data and methods

### 2.1 Study area and data sources

In this study, 2869 counties in 31 Chinese provincial administrative units (excluding Taiwan Province, Hong Kong Special Administrative Region, and Macao Special Administrative Region) are selected as the study units, given their possession of high-quality data and extensive coverage, which can adequately reflect the status and characteristics of lung cancer incidence in China. To account for the diverse physical and geographical characteristics of China, we divide the country into seven divisions (Table 1).

**Table 1:** Seven geographical divisions of China.

Geographical Division	Region
Northeast China	Heilongjiang, Jilin, Liaoning and Hulunbeier City, Xing an League, Tongliao City, Chifeng City and Xilinguole League in eastern Inner Mongolia
East China	Shandong, Jiangsu, Shanghai, Zhejiang, Anhui, Jiangxi and Fujian
North China	Hebei, Beijing, Tianjin, Shanxi, and Part of Inner Mongolia
Central China	Henan, Hubei, Hunan
South China	Guangdong, Guangxi, Hainan
Southwest China	Sichuan, Yunnan, Guizhou, Chongqing, Tibet
Northwest China	Xinjiang, Ningxia, Qinghai, Shaanxi and Gansu

We obtained the lung cancer incidence data from the Cancer Atlas in China (2018), which is compiled by the National Cancer Center/Cancer Hospital Chinese Academy of Medical Sciences. This database encompasses China’s tumor registry data, cause-of-death surveillance information in 2014, and specific cancer-related explanatory variables. Spatial analysis models are used to obtain the cancer incidence data in each district and county in China, classified into 10 levels. The atlas clarify China’s geographical distribution of major cancers. As for the baseline data of Chinese county-level units, we procure them from the 2010 Chinese county-level administrative divisions.

Data on factors influencing lung cancer incidence are obtained through different pathways, while the PM2.5 concentration data are retrieved from the Atmospheric Composition Analysis Group of Dalhousie University (http://fizz.phys.dal.ca/~atmos/martin/?page_id=140); the temperature, precipitation, atmospheric pressure, and elevation data were acquired from the *Daily Surface Climate Dataset for China (V3*.*0)* in the China Meteorological Data Service Center (http://data.cma.cn/). This study matches these spatial meteorological data with the administrative data from counties and districts. To mitigate biases arising from years with extreme weather conditions, we calculate the average values of these five atmospheric and environmental factors for the period 2010–2014.

Data sources for the influencing factors of the control variables (socio-economic characteristics), including population density, urbanization rate, and education years per capita of the population, are sourced from the Tabulation on the 2010 Population Census of the People’s Republic of China by County. Furthermore, the population density is calculated by dividing the number of residents by the area of the county administrative district, while the urbanization rate is derived by dividing the urban population by the total resident population. Additionally, the average wages in urban areas are retrieved from various sources, including the China City Statistical Yearbook, China County Statistical Yearbook, and Statistical Yearbook for the Regional Economy. We opt to utilize socio-economic characteristics data from 2010 as they tend to be relatively stable in the short term.

### 2.2 Spatial autocorrelation analysis and hotspots exploration

The global Moran’s *I* is employed to detect the overall spatial characteristics of lung cancer incidence, and it is expressed as follows [32, 33]:

$$I=\frac{\sum_{i=1}^{n}\sum_{j=1}^{n}W_{ij}\left(x_{i}-\bar{x}\right)\left(x_{j}-\bar{x}\right)}{S^{2}\sum_{i=1}^{n}\sum_{j=1}^{n}W_{ij}}$$ (1)

$$S^{2}=\frac{\sum_{i=1}^{n}{\left(x_{i}-\bar{x}\right)}^{2}}{n}$$ (2)

where *I* is the global spatial autocorrelation index; *x*_*i*_ and *x*_*j*_ represent the lung cancer incidence of the *i*th and *j*th county, respectively; and *W*_*ij*_ is the weight matrix of each county. The value range of *I* is [–1, 1]. *I* > 0, lung cancer incidence is positively correlated; conversely, it shows a negative correlation. *Z* is the standardized statistic of *I*, which is used to determine the degree of lung cancer incidence agglomeration. The value of *Z* can be expressed as:

$$Z\left(I\right)=\frac{I-E\left(I\right)}{\sqrt{Var\left(I\right)}}$$ (3)

where *Var(I)* represents the variance, and *E(I)* is the mathematical expectation of lung cancer incidence. When the absolute value of *Z* is high, it indicates that the positive (negative) spatial correlation of the lung cancer incidence is more significant, and the absolute value of *Z* tends to 0, which means that the result is not significant, and the lung cancer incidence is randomly distributed.

### 2.3 Spatial regression analysis

Lung cancer incidence data are age-standardized using Segi’s world standard population to obtain world age-standardized incidence rates (ASR world) in 1/100000 and divided into 10 classes in the following order: <15, 15–21, 21–27, 27–32, 32–36, 36–40, 40–45, 45–52, 52–63, and 63–93 (1/100000) to make a Chinese ASR world incidence distribution map [34]. Based on this map, we manually digitize the data to obtain lung cancer incidence in each county in China.

By sorting out and summarizing existing studies, we select nine influencing factors of lung cancer incidence from two perspectives: atmospheric environment and socio-economic characteristics (Table 2). Regarding the atmospheric environment, we choose five factors as explanatory variables, including PM2.5 concentration, temperature, precipitation, atmospheric pressure, and elevation. Regarding socio-economic characteristics, we identify four factors as control variables, including population density, urbanization rate, wages, and education years per capita. The correlation between these socio-economic factors and lung cancer incidence has been demonstrated [35–38]. Data used in this study can be obtained from http://39.108.95.102:2233/team.html.

**Table 2:** Descriptive statistics on factors influencing lung cancer incidence.

Variable	Unit	Min	Max	Mean	Std.Dev	Expected direction
**Explanatory Variables—Atmospheric environment characteristics**						
PM2.5 concentration	μg/m^3^	1.0512	109.8966	47.7015	24.5996	+
Temperature	°C	0.7898	24.2696	13.4534	4.6540	-
Precipitation	mm	19.8000	2817.6800	914.8808	503.7726	+
Atmospheric pressure	hPa	569.1800	1016.8400	950.9656	88.7174	+
Elevation	m	1.8000	4800.0000	594.2279	867.4332	-
**Control Variables—Socio-economic characteristics**						
Population density	person/sq.km	0.1263	72,680.0871	1373.3175	4274.4084	+
Urbanization rate	%	1.3796	100.0000	46.9149	25.6777	+
Wages	10,000 yuan	0.5129	12.5949	3.0861	0.9614	+
Education years per capita	year	2.0000	13.1400	8.7140	1.4672	+

We use ArcGIS software (Version 10.7) to analyze spatial autocorrelation of China’s lung cancer incidence, and SPSS software (Version 22) to calculate variance inflation factor (VIF) values for covariance diagnosis between independent variables, with VIF < 10 considered as no covariance between variables and reasonable variable selection. Furthermore, we construct regression models using the GeoDa software, which can be obtained from the website of the Center for Spatial Data Science, the University of Chicago (Version 1.18, https://spatial.uchicago.edu/software). The GeoDa software supports the development of three regression models: ordinary least squares (OLS), spatial lag model (SLM), and spatial error model (SEM).

OLS is a traditional linear regression model that does not examine the interaction between adjacent county variables, whereas SLM and SEM are spatial econometric models. The equation is as follows [39]:

$$y_{i}=\beta X_{i}+\varepsilon_{i},\left[\varepsilon_{i}\sim N(0,\delta^{2}I)\right]$$ (4)

Where *y*_*i*_ denotes the lung cancer incidence in the *i*th county (*i* = 1, 2, 3…, 2869), is the independent variable of the mode; *x*_*i*_ the *s*-dimensional row vector of influencing factors on lung cancer incidence (*s* = 1, 2,…, 9), denoting the value of the *s*th influencing factor in the ith county; *β* is the *s*-dimensional row vector, denoting the regression coefficients of the 9 influencing factor; *ε* is the random error term of the model, *i*~*N*(0, *δ*^*2*^*I*) denoting that the error term must obey a normal distribution, and *I* is the unit matrix.

SLM analyzes the spatial interactions of lung cancer incidence in neighboring counties and if the lung cancer incidence in a county strongly correlates with that in the surrounding counties. Conversely, SEM deals with the spatial dependence effect through the spatial autocorrelation setting of the error terms. The following equation expresses the model [40, 41]:

$$y_{i}=\rho \sum_{j=1}^{n}W_{ij}y_{j}+\beta X_{i}+\varepsilon_{i},\left[\varepsilon_{i}\sim N(0,\delta^{2}I)\right]$$ (5)

Where *I* represents the value of spatial autocorrelation coefficient; *W*_*ij*_ stands for spatial weight matrix.

SEM can be used when unobservable variables have spatial spillover effects. The equation is expressed as follows [42]:

$$y_{i}=\lambda \sum_{j=1}^{n}W_{ij}\varphi_{i}+\beta X_{i}+\varepsilon_{i},\left[\varepsilon_{i}\sim N(0,\delta^{2}I)\right]$$ (6)

In Eq (6), *ϕ* is the spatially autocorrelated error term in the model, and *λ* is the spatial autocorrelation coefficient of the random error term.

The optimal model is selected based on a combination of Moran’s *I* (error), Lagrange Multiplier (LM), Robust Lagrange Multiplier, *R*^*2*^, Akaike information criterion (AIC), and log-likelihood parameters to determine whether atmospheric environment factors affect lung cancer incidence, with the magnitude and direction of the effect. Before running these three regression models, we performed log standardization of all independent variables to eliminate the magnitude effect.

## 3. Results

### 3.1 Spatial variation and spatial autocorrelation characteristics of the lung cancer incidence in China

**Fig 1:**
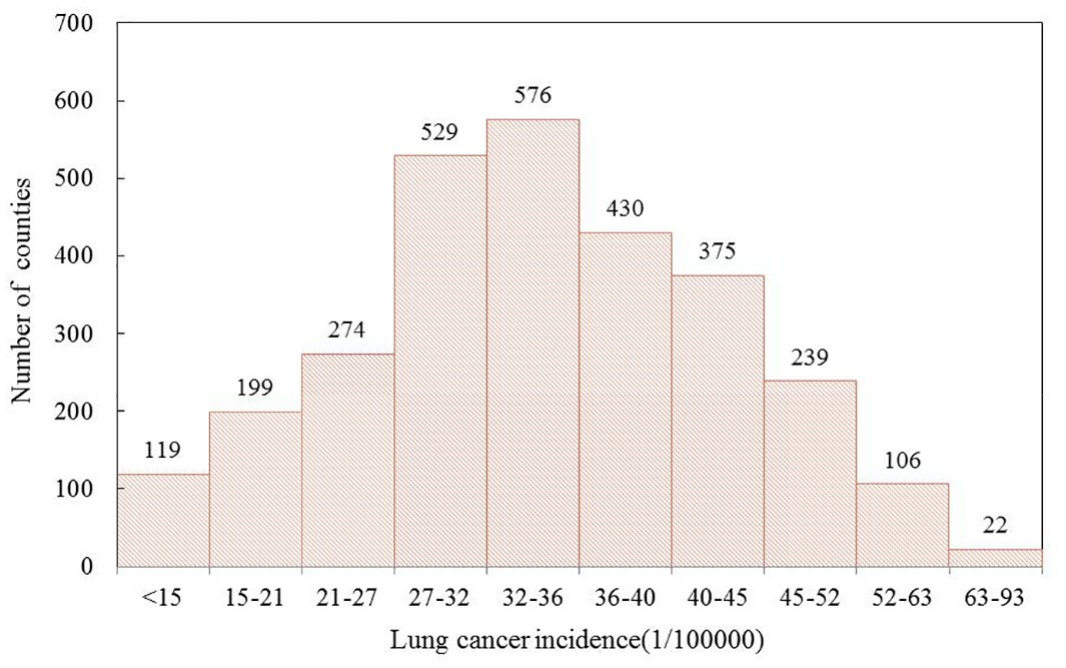
Number of counties with different levels of lung cancer incidence.

Based on the data obtained in this study, we conduct a count of county units according to different levels of lung cancer incidence, as depicted in Fig 1. Among them, there are 576 counties with lung cancer incidence ranging from 32–36 (1/10000), which accounts for the highest number of units at 20.08% of the total counties. 22 counties with lung cancer incidence rates of >63 (1/10000) have the lowest number of units, and counties in this range have the highest proportion of people with lung cancer, and the most serious lung cancer incidence, which requires more attention. According to the National Central Cancer Registry, lung cancer incidence in China in 2014 was approximately 36.63/100000, with 1172 counties having an incidence greater than 36/100000, accounting for 40.85% of the total county-level units, indicating the severity of lung cancer incidence in China.

To conduct an in-depth analysis of the spatial uneveness in lung cancer incidence across China’s seven major geographical divisions, we have compily statistics on the number of counties with different levels of lung cancer incidence (Table 3). There are 288 counties with lung cancer incidence higher than 36/100,000 in the Northeast China region, accounting for 87% of the total number of counties, among which 10 counties (All 22 counties in China) have lung cancer incidence significantly higher than 63/100,000, mainly concentrated in Yingkou and Liaoyang in Liaoning Province, and Harbin, Daqing, Qiqihaer, and Jiamusi in Heilongjiang Province, which fully reveals that the severe situation of lung cancer incidence in Northeast China; Comparatively speaking, the situation in East China (331 counties), North China (387 counties) and Northwest China (360 counties) is relatively optimistic, with 339, 257 and 202 counties with lung cancer incidence lower than 36/100,000, accounting for 52.8%, 66.41% and 91.11%, respectively, which indicates that the lung cancer incidence in these regions are relatively low; in Central China, the number of counties with lung cancer incidence lower than 36/100000 is 202, accounting for 52.6%, while 2 counties with lung cancer incidence more than 63/100000 are Lushan County and Yuzhou City in Henan Province; in Southwest China (511 counties), the number of counties with lung cancer incidence lower than 36/100000 is 383, accounting for 74.95%, of which lung cancer incidence in Qilin District of Yunnan Province and Luxian County of Sichuan Province are higher than 63/100,000; in South China, there are 186 counties with lung cancer incidence lower than 36/100,000, accounting for 73.22% of the total, but it is worth noting that there are 8 counties with high incidence (>63/100,000) (All 22 counties in China), concentrating on Shenzhen and Zhanjiang in the coastal area of Guangdong Province and Nanning in Guangxi. Overall, the lung cancer incidence is relatively high in Northeast China, while some areas of high lung cancer incidence still exist in Central China, Southwest China and South China, although the overall lung cancer incidence is relatively low.

**Table 3:** Number of counties with different levels of lung cancer incidence in seven geographical divisions of China.

Geographical Division	Lung cancer incidence(1/100000)									
	<15	15–21	21–27	27–32	32–36	36–40	40–45	45–52	52–63	63–93
Northeast China	0	3	4	9	27	36	102	95	45	10
East China	0	3	37	143	156	121	102	59	21	0
North China	5	5	40	106	101	64	36	15	15	0
Central China	3	6	22	65	106	99	42	29	10	2
South China	1	5	13	49	77	52	29	14	6	8
Southwest China	79	64	79	83	78	49	49	21	7	2
Northwest China	31	113	79	74	31	9	15	6	2	0

We calculate Moran’s *I* value to test whether there is a spatial autocorrelation in lung cancer incidence, which may have spatial correlation characteristics in Chinese counties. The Moran’s *I* value is 0.7593, with a *z*-score of 68.6769 and *p*-value of 0.0000, indicating that lung cancer incidence in Chinese counties has strong spatial autocorrelation and correlation. That is, there is a correlation between lung cancer incidence in a county and its neighboring counties.

The results reveal that lung cancer incidence in China has obvious geospatial unevenness and large regional variation, indicating the need for more attention on priority areas for lung cancer prevention and treatment.

### 3.2 Influence of atmospheric environment on lung cancer incidence

**Fig 2:**
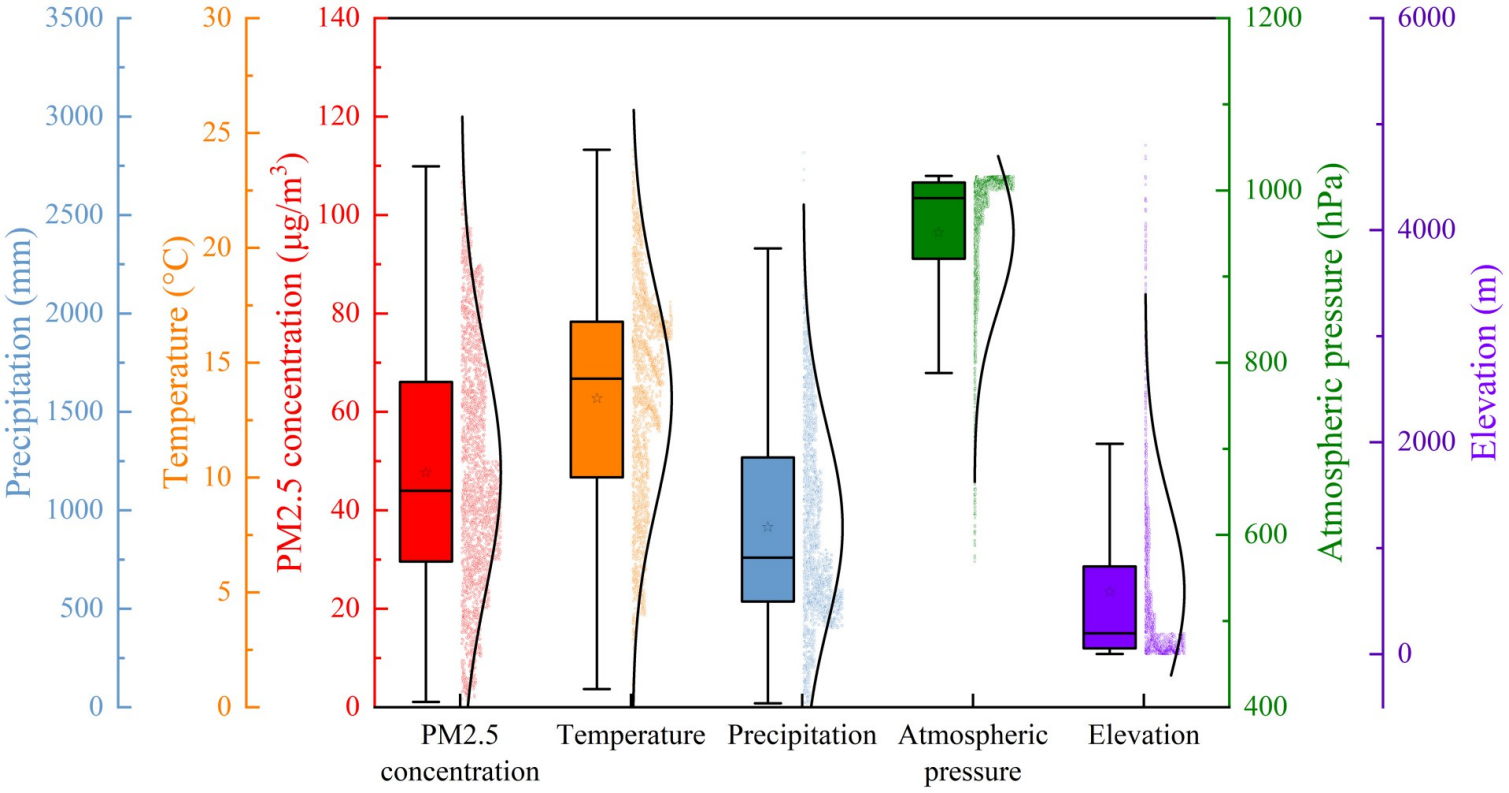
Boxplot of distribution of five atmospheric environment factors.

Fig 2 shows the boxplot of the data distribution of the five atmospheric environment factors, which visually illustrates the distribution characteristics of the data.

Lung cancer incidence exhibits a correlation with geographical location; however, geographic location is not the only cause of lung cancer incidence. Rather, the hidden atmospheric environment spatial variation behind different geographic areas is the real cause of the variation in lung cancer incidence. Therefore, we conduct the factors influencing lung cancer incidence, intensity, and direction.

Firstly, we employ the Pearson Correlation Coefficient to investigate the correlation between the PM2.5 concentration and atmospheric environment parameters. Table 4 reveals a significant correlation between PM2.5 concentration and the atmospheric environment, thus providing robust evidence for the association between lung cancer and atmospheric conditions.

**Table 4:** Correlation between PM2.5 concentration and atmospheric parameters.

	Temperature	Precipitation	Atmospheric pressure	Elevation
Correlation (partial correlation coefficient)	0.514***	0.151***	0.636***	-0.491***
Significance (two-sided test)	0.000	0.000	0.000	0.000

Note:

***,**,* indicate significance at 0.01, 0.05, and 0.1 levels, respectively

Then, we test the variables for covariance using SPSS software to verify the rationality of the selected variables. The results, as displayed in Table 5, demonstrate that the VIF (Variance Inflation Factor) values for the nine independent variables are all below 10, indicating the absence of covariance among these variables. This finding substantiates the appropriateness of including these independent variables in the regression model.

**Table 5:** Covariance diagnosis of variables.

Variable	Tolerance	VIF
Intercept	—	—
PM2.5 concentration	0.297	3.370
Temperature	0.402	2.487
Precipitation	0.419	2.389
Atmospheric pressure	0.207	4.829
Elevation	0.374	2.672
Urbanization rate	0.294	3.400
Wages	0.289	3.457
Education years per capita	0.676	1.480
Urbanization rate	0.228	4.386

We also use GeoDa software to determine the optimal regression model. We ran the OLS model with weights set to Queen and performed Moran’s *I* test. The results show that Moran’s *I* (error) is 0.5906, the *z*-value is 50.9338 and the *p*-value is 0.0000, indicating that the model residuals have spatial autocorrelation and spatial regression models, SLM and SEM, should be considered. Subsequently, we compare the LM and Robust LM values of SLM and SEM, both of which are significant with *p* <0.01. We then compare these models’ *R*^2^, log-likelihood, and AIC values(Table 6). SEM has the highest *R*^2^ and log-likelihood values and the lowest AIC value, indicating that SEM provides the best fit. Thus, we select SEM to analyze factors influencing lung cancer incidence in Chinese counties.

**Table 6:** Comparison of main parameters of three regression models.

Model	*R* ^2^	AIC	Log likelihood
OLS	0.457749	10418.9	-5199.45
SLM	0.736966	8620.69	-4299.35
SEM	0.776107	8352.93	-4166.463204

The results of SEM model are shown in Table 7. Table 7 shows that four atmospheric environment factors significantly affect lung cancer incidence, namely PM2.5 concentration, temperature, atmospheric pressure, and elevation. The coefficient of PM2.5 concentration is positive and significant at the 0.01 level, indicating that PM2.5 concentration plays a role in promoting lung cancer incidence. A 1% increase in PM2.5 concentration is associated with a level of 0.002975 increase in lung cancer incidence. A strong relationship between PM2.5 and lung cancer incidence has long been confirmed [23, 43, 44]. PM2.5 refers to small respirable particles with a diameter of less than or equal to 2.5 μm. Prolonged exposure to high concentrations of PM2.5 allows these particles to enter the human respiratory tract and come into close contact with lung cells, damaging them by inducing apoptosis. This prompts the lungs to undergo active cell division to replenish the damaged cells, a process that can increase the risk of lung cell mutation and ultimately lead to a higher incidence of lung cancer. Temperature increase may hinder lung cancer incidence and have the most significant effect when compared to other atmospheric environment factors. A 1% increase in temperature is associated with a level of 0.006443 decrease in lung cancer incidence. Temperature may indirectly affect lung cancer incidence because it affects people’s lifestyles, especially in cold regions where people must burn coal for heating, a process that generates many harmful gases. Moreover, low temperatures are not conducive to the diffusion of air pollutants such as PM2.5 and PM10, leading to increased lung cancer.

**Table 7:** Regression coefficients of influencing factors based on SEM model.

Variables	Coefficients	Std.Error	*z*-value	*p*
Constant	-14.7616***	4.7417	-3.1132	0.0019
PM2.5 concentration	0.2975***	0.1092	2.7235	0.0065
Temperature	-0.6443***	0.1707	-3.7743	0.0002
Precipitation	0.0877	0.0875	1.0020	0.3164
Atmospheric pressure	2.6061***	0.6971	3.7385	0.0002
Elevation	-0.0934**	0.0369	-2.5283	0.0115
Population density	0.1211***	0.0264	4.5800	0.0000
Urbanization rate	0.2540***	0.0639	3.9762	0.0001
Wages	0.1702	0.1054	1.6151	0.1063
Education years per capita	0.2664	0.2558	1.0415	0.2977
LAMBDA	0.8188***	0.0118	69.1054	0.0000
*R*^*2*^: 0.776107; AIC: 8352.93; Log likelihood: -4166.463204				

Note:

***,**,* indicate significance at 0.01, 0.05, and 0.1 levels, respectively

At the significant level of 0.01, atmospheric pressure positively affects lung cancer incidence, with a 1% increase in atmospheric pressure leading to a 0.026061 increase in lung cancer incidence. Atmospheric pressure may also indirectly influence lung cancer by changing meteorological factors such as temperature and precipitation, affecting air pollutants’ diffusion to increase lung cancer risk. The elevation coefficient is negative and significant at the 0.05 level. A negative correlation exists between elevation and lung cancer incidence, where an increase in elevation by 1% correlates with a decrease in lung cancer incidence by a level of 0.000934. The higher the elevation, the thinner the air, and the lower the oxygen concentration, the lower the risk of lung cancer [30]. Moreover, higher elevations correlate with fewer carcinogenic pollutants in the air and more sunlight, which increases vitamin D absorption and helps prevent lung cancer. The regression model results indicate that precipitation had no significant effect on lung cancer incidence.

The other four control variables reflect the effect of socio-economic factors on lung cancer incidence. At the significance level of 0.01, both population density and urbanization rate have significant effects on lung cancer, with higher population density and urbanization rates correlating with increased lung cancer incidence. Specifically, a 1% increase in either population density or urbanization rate is associated with an increase in lung cancer incidence by 0.001211 or 0.00254, respectively. In contrast, the relationship between lung cancer incidence and both wages and the education years per capita of the population is insignificant. This finding is consistent with Lin et al., who discovered that lung cancer has a high incidence in people with high incomes and residences [38].

We conduct regression analysis using the GeoDa software for the seven geographical divisions in China to explore the impact of the atmospheric environment on lung cancer incidence in various areas. After comparing the three models, the SEM emerge as the most suitable model. According to Table 8, in Northeast China, temperature plays a crucial role in lung cancer incidence. At a significance level of 0.01, a 1% increase in temperature is associated with a decrease of 0.007425 in lung cancer incidence. In the East China region, there is a significant negative correlation between precipitation and lung cancer incidence. For every 1% increase in precipitation, lung cancer incidence decreases by 0.011288 (p<0.01). In the North China region, the atmospheric environment has a significant impact on lung cancer incidence. PM2.5 concentration, temperature, precipitation, and elevation all contribute to the incidence. At a significance level of 0.01, a 1% increase in PM2.5 concentration is associated with a 0.009888 increase in lung cancer incidence, while a 1% increase in elevation is associated with a decrease of 0.003271 in lung cancer incidence. At a significance level of 0.05, a 1% increase in temperature is associated with a decrease of 0.023881 in lung cancer incidence. Furthermore, at a significance level of 0.1, a 1% increase in precipitation leads to a decrease of 0.007036 in lung cancer incidence. Among the five factors of the atmospheric environment, only PM2.5 concentration has a significant impact on lung cancer incidence in the Central China region. At a significance level of 0.05, a 1% increase in PM2.5 concentration results in a 0.016791 increase in lung cancer incidence. In South China, PM2.5 concentration and temperature are significantly correlated with lung cancer incidence. A 1% increase in PM2.5 concentration leads to a 0.016791 increase in lung cancer incidence (p<0.05), while a 1% increase in temperature is associated with a decrease of 0.056157 in lung cancer incidence (p<0.1). In the Southwest China region, only PM2.5 concentration has a significant impact on lung cancer incidence. At a significance level of 0.01, a 1% increase in PM2.5 concentration is associated with a 0.005486 increase in lung cancer incidence. In the Northwest China region, there is a significant positive correlation between lung cancer incidence and precipitation. For every 1% increase in precipitation, lung cancer incidence increases by 0.002664. In summary, the intensity and direction of the impact of the atmospheric environment on lung cancer incidence vary across different regions.

**Table 8:** The influence intensity of atmospheric environment in different geographical divisions on lung cancer incidence on SEM model.

Geographical Division	Northeast China	East China	North China	Central China	South China	Southwest China	Northwest China
Constant	8.2541	16.7940	32.9986	-38.6682	-19.2601	-10.0217	-8.8418
	(0.5935)	(0.6199)	(1.3113)	(-0.8175)	(-0.4294)	(-1.0408)	(-0.973)
PM2.5 concentration	0.4656	0.1720	0.9888***	1.6791**	-1.3099**	0.5486***	0.2604
	(0.1490)	(0.5437)	(2.7790)	(2.4276)	(-2.2404)	(2.9564)	(1.1607)
Temperature	-0.7425***	-0.8797	-2.3881**	0.0498	-5.6157*	-0.1290	0.3967
	(-2.7248)	(-0.8622)	(-2.4469)	(0.0242)	(-1.9375)	(-0.2693)	(0.7731)
Precipitation	-0.1758	-1.1288***	-0.7036*	-0.2708	-0.0275	0.1810	0.2664*
	(-0.9183)	(-3.2150)	(-1.8458)	(-0.5204)	(-0.0498)	(0.9110)	(1.8568)
Atmospheric pressure	0.3825	-0.3969	-3.2644	5.2613	5.4343	1.3713	0.6787
	(0.1957)	(-0.1002)	(-0.8966)	(0.7369)	(0.8229)	(0.9603)	(0.5173)
Elevation	-0.0926	0.0428	-0.3271***	0.0920	-0.1162	-0.0573	-0.0238
	(-0.7969)	(0.6375)	(-2.8845)	(0.5159)	(-0.8845)	(-0.4159)	(-0.1656)
Population density	0.1466**	0.0524	-0.0056	0.0018	0.4066***	0.3207***	0.1459**
	(2.1417)	(0.8000)	(-0.0714)	(0.0148)	(3.3634)	(4.3409)	(2.2808)
Urbanization rate	0.1698	0.3308*	0.0963	0.1519	0.2644	0.2586**	0.1076
	(0.8844)	(1.7303)	(0.3930)	(0.5925)	(1.0434)	(2.4649)	(0.7267)
Wages	-0.0424	0.2369	0.0235	0.2725	-0.0542	0.3580	0.4712
	(-0.1819)	(0.9852)	(0.0810)	(0.7508)	(-0.1497)	(1.2075)	(1.5136)
Education years per capita	-1.6810	-0.3969	0.8585	0.5030	2.8458**	-0.1449	1.3159**
	(-1.5838)	(-0.5674)	(0.6903)	(0.478)	(2.1677)	(-0.3806)	(2.2038)
LAMBDA	0.7189***	0.7664***	0.7075***	0.7192***	0.6708***	0.7649***	0.6880***
	(17.5169)	(27.9644)	(16.6603)	(17.6877)	(12.6494)	(22.9216)	(15.2265)
R^2^	0.588834	0.66709	0.566807	0.574893	0.599876	0.842806	0.652652
AIC	969.365	1792.02	1200.55	1164.24	793.933	1380.44	1055.55
Log Likelihood	-474.6824	-886.0817	-590.2747	-5720.1216	-386.9666	-680.2214	-517.7735

Note: *t*-statistics in parentheses,

***, **, * represent the 0.01, 0.05, and 0.1 significance levels, respectively.

## 4. Discussion

This study investigates the spatial variation of lung cancer incidence and the factors influencing lung cancer incidence from the perspective of the atmospheric environment based on lung cancer incidence data from the National Cancer Center/Cancer Hospital Chinese Academy of Medical Sciences, which compiled the Cancer Atlas in China (2018). As one of the few studies on this research topic, this study’s findings could provide guidance and reference information for lung cancer prevention and management in China and other countries.

Firstly, we present the spatial variation and spatial agglomeration of lung cancer incidence in Chinese county-level units and discover that lung cancer incidence in China exhibits significant geographical unevenness. Overall, the lung cancer incidence is relatively high in Northeast China, while some areas of high lung incidence still exist in Central China, Southwest China and South China, although the overall lung cancer incidence is relatively low. In particular, the Northeast region stands out as a notable area with a pronounced prevalence of lung cancer. The origins of this issue are likely multifaceted. Firstly, as a former industrial hub of China, the Northeast region is home to numerous heavy industrial enterprises and mineral resources. These industrial operations can generate a considerable amount of air pollutants, including PM2.5, SO_2_, NO_x_, and others. Prolonged exposure to these pollutants can significantly increase the risk of developing lung cancer. Secondly, the long and cold winters in the Northeast region often prompt residents to restrict indoor and outdoor air circulation for warmth, compromising indoor air quality. The accumulation of harmful fumes from tobacco smoke, coal combustion for heating, and noxious substances emitted by building materials, such as formaldehyde, poses a constant threat to lung health. Additionally, the Northeast region heavily relies on coal-fired heating stations for warmth in winter. This process results in the emission of significant amounts of pollutants, which not only severely affect air quality but also penetrate deep into the lungs. Prolonged exposure to these pollutants can lead to respiratory damage, genetic mutations, and ultimately contribute to the occurrence of lung cancer. These combined factors collectively contribute to the increasing lung cancer incidence in the Northeast region. The display of spatial variation and characteristic analysis can indicate a relationship between lung cancer incidence and the atmospheric environment, laying the foundation for further exploration of factors influencing lung cancer incidence. In addition, displaying the spatial variation of lung cancer incidence provides a clear reference for relevant departments to formulate precise lung cancer prevention and treatment policies while balancing medical resources.

Our findings demonstrate that SEM is the optimal model for studying the atmospheric environment and lung cancer incidence, among the three models used in this study. This provides a useful source of study approaches for other researchers and indicates that lung cancer incidence in a county is influenced by the atmospheric environment of that county and those of its surrounding counties. This implies that the atmospheric environment factors of lung cancer incidence have spatial spillover effects. Based on SEM, PM2.5 concentration and atmospheric pressure positively affect lung cancer incidence, while temperature and elevation have the opposite effect. This is consistent with previous studies [23, 29, 30, 45, 46], which have shown that PM2.5 is involved in promoting lung cancer initiation, growth, and progression, and stimulating cancer cell proliferation, migration, and invasion [45]. In addition, this study demonstrates the promoting effect of atmospheric pressure on lung cancer incidence, which has rarely been reported in the literature, suggesting that atmospheric pressure should be considered in future studies. Some studies have also reported that meteorology is an important factor affecting air pollutants [47]. Temperature, as a meteorological factor, plays a key role in the diffusion and accumulation of air pollutants, thus indirectly influencing lung cancer incidence. It has been reported that altitude is significantly and negatively correlated with lung cancer incidence [29, 30]. The role of altitude in lung cancer is manifested in two-fold: on the one hand, increasing altitude reduces the oxygen content of the atmosphere; on the other hand, the pollutant concentration in the air decreases, and light intensity increases.

In addition, China is geographically vast with diverse environmental conditions, and the geographical environment has different effects on lung cancer incidence across regions. The frigid climates of Northeast China make low temperatures a significant determinant of lung cancer incidence. Conversely, the East China region enjoys abundant rainfall, and the "washing effect" of precipitation helps to remove pollutants such as PM2.5, PM10, sulfur compounds, and nitrogen compounds from the air. This may contribute to lower lung cancer incidence. In North China, notably in urban hubs like Beijing, Tianjin, and Hebei, pervasive industrialization, widespread coal consumption, and intense vehicular activity conspire to elevate PM2.5 levels. Altitudinal discrepancies between regions like Shanxi and Inner Mongolia also modulate pollutant concentrations, as high-altitude locales experience air rarefaction, attenuating certain pollutants. Additionally, the rigors of winter and the scorching summers influence indoor heating practices and daily routines, thus modulating respiratory exposures. The Central China region is mainly composed of low to medium mountains and vast plains. The relatively low-lying and flat topography is not conducive to the dispersion of air pollutants, especially under weak wind conditions, where pollutants tend to accumulate near the ground. In lower-altitude areas, the higher air density may lead to relatively higher concentrations of pollutants. These geographical and climatic factors interact with each other, potentially resulting in higher PM2.5 concentrations in the Central China region, thereby increasing the risk of lung cancer among residents. For instance, Wei et al. found in their study on lung cancer incidence in Henan Province that due to its inland location and flat terrain in the eastern part, air pollutants cannot disperse and accumulate there, making PM2.5 concentration the main cause of lung cancer incidence [5]. In the South China region, industrial development, vehicle emissions, and high population density often lead to higher concentrations of PM2.5. The hot weather encourages residents to engage in indoor activities, reducing their exposure to external air pollution. Additionally, high temperatures may also alter the distribution and chemical transformation rates of air pollutants. In the Southwest China region, cities such as Chongqing and Chengdu have experienced rapid industrial development and a surge in vehicles. Coupled with mountainous and basin terrain, this may result in the accumulation of air pollutants in certain areas, particularly in basins with poor air circulation, making it difficult for pollutants to disperse and leading to higher local PM2.5 concentrations. In the Northwest China region, the lack of rainfall due to arid conditions prevents the timely cleansing of air pollutants.

The present study takes into account the spatial interactions of lung cancer development in neighboring regions. The study covers 2869 county-level units in China with complete and detailed information, providing a comprehensive presentation of the spatial variation and characteristics of lung cancer incidence and influencing factors in China. The spatial regression model considering spatial interactions in neighboring regions increases the credibility and applicability of our findings. These results can be applied to other countries or regions and etiology studies of other types of diseases.

Unfortunately, this study has some limitations. First, it only considered spatial variation in lung cancer incidence, without taking into account temporal differences. Moreover, environmental factors can affect lung cancer risk in men and women, and thus, future discussions should separately address these groups. Second, this study only focused on nine influencing factors, mainly five atmospheric environment factors, while lung cancer is caused by a combination of various factors. Therefore, we should consider additional influencing factors such as wind speed, humidity, and light intensity in future studies. Third, this study did not consider the spatial heterogeneity of the relationship between lung cancer incidence and atmospheric environment. In recent years, China’s accelerated urbanization, population aging, and socio-economic development may have increased the cancer disease burden and led to changes in cancer status [48]. In the future, we can focus on the role of social factors in lung cancer incidence.

Lung cancer incidence and mortality are rising rapidly in China. The Global Cancer Statistics 2020 showed that approximately 715,000 deaths from lung cancer occurred in China. Over the past 40 years, lung cancer mortality in China has increased nearly four-fold, accounting for 27.3% of all cancer deaths. In recent years, lung cancer incidence has decreased yearly in many countries due to the implementation of tobacco control campaigns. On the contrary, lung cancer incidence in China is still increasing. The establishment of China Anti-lung Cancer Alliance aims to effectively reduce lung cancer incidence and mortality. The Chinese Alliance Against Lung Cancer was established to effectively reduce lung cancer incidence and mortality. This organization has joined several medical forces involved in lung cancer prevention and treatment to promote early diagnosis and comprehensive lung cancer treatment and to prolong the lives of patients with lung cancer. The Rural Cancer Early Diagnosis and Treatment Program and the Urban Cancer Early Diagnosis and Treatment Program, supported by the Chinese government, have officially been included in the National Major Public Health Service Project. This project aims to provide free screening, early diagnosis, and treatment for highly prevalent cancers, including lung cancer, to reduce the financial burden for patients. Early screening for breast, cervical, and colorectal cancers has been implemented in the United Kingdom and the United States, contributing to the decrease in cancer incidence and mortality [49, 50], justifying the need for universal early cancer screening. Inadequate early lung cancer screening coverage is a challenge, which constrains the reduction of lung cancer incidence. Therefore, based on this study, we should enhance early screening for lung cancer in areas with high-risk atmospheric environment factors.

Therefore, it is recommended that people living in areas with high-risk atmospheric environment factors, long-term smokers, individuals with a family history of lung cancer, and those frequently exposed to oil, smoke, or soot; as well as those residing in areas with severe air pollution for extended periods, and those at greater risk of developing lung cancer, should undergo annual low-dose spiral computed tomography screening. This method can detect tumors earlier and more accurately than lung x-ray screening and radiographs. Through early lung cancer screening, the survival cycle and treatment rate of early-stage patients can be effectively improved. In regions with higher incidence rates, relevant authorities can assist in redirecting medical resources to improve the treatment of lung cancer and reduce mortality.

The atmospheric environment and lung cancer incidence are highly correlated, and the cancer risk factors underlying atmospheric environment variation are often controllable. Moreover, many of these cancer-causing risks may be avoidable by evading adverse environments, adopting healthier lifestyles, and taking effective measures. For instance, people living in high-risk geographic areas can reduce their risk of lung cancer by regularly convalescing in low-risk areas. The government and administrators should take measures to promote regional coordinated development, address geographical imbalances, optimize the spatial allocation of medical resources, improve medical care levels, reduce taxes and fees for importing special drugs, and ease the financial and mental burden on patients with cancer. It is essential to raise awareness of lung cancer prevention and strive for "early detection, diagnosis, and treatment". In recent years, more patients with cancer have been able to afford treatment due to the expansion of medical insurance services in China, the improvement of reimbursement policies, and the reduction of anti-cancer drug prices.

## 5. Conclusions

Lung cancer incidence in China has apparent changes in geographical and spatial unevenness and spatial autocorrelation, characteristics. In China, higher lung cancer incidence rates are mainly located in the Northeast region, while Central China, Southwest China, and South China, although having generally lower lung cancer incidence, still have some regions with higher incidence.

The atmospheric environment has a significant impact on lung cancer incidence in China. PM2.5 concentration, temperature, atmospheric pressure, and elevation affect lung cancer incidence. Different elements of the atmospheric environment vary in the direction and extent of their influence on the development of lung cancer. At the 0.01 significant level, PM2.5 concentration, and atmospheric pressure positively affect lung cancer incidence, and temperature negatively affects lung cancer incidence; at the 0.05 significant level, elevation has a significant negative effect on lung cancer incidence. In contrast, atmospheric pressure has the strongest effect on lung cancer incidence. Moreover, the influence intensity and direction of the atmospheric environment vary from one region to another in terms of their impact on lung cancer incidence.

Governments and the public should actively address lung cancer for early prevention to reduce the burden of lung cancer effectively. In high-risk geographic areas, the government should intensify the dissemination of lung cancer prevention knowledge, raise public awareness of cancer, and motivate the public to undergo early lung cancer screening to achieve early detection, diagnosis, and treatment, which will effectively reduce lung cancer incidence and mortality. People living in high-risk geographic areas can regularly travel to low-risk geographic areas for convalescence. Healthcare policymakers should incorporate the influence of the atmospheric environment into regulations to reduce lung cancer incidence.

## Supporting information

S1 Data (XLS)
